# Nitrogen-Doped Carbon Nanosheets Decorated With Mn_2_O_3_ Nanoparticles for Excellent Oxygen Reduction Reaction

**DOI:** 10.3389/fchem.2019.00741

**Published:** 2019-11-07

**Authors:** Maryam Qasim, Jianhua Hou, M. A. Qadeer, Sajid Butt, M. Hassan Farooq, M. Qasim Farooq, Faryal Idrees, M. Tanveer, Jijun Zou, Muhammad Tahir

**Affiliations:** ^1^Department of Physics, The University of Lahore, Lahore, Pakistan; ^2^School of Environmental Science and Engineering, Yangzhou University, Yangzhou, China; ^3^Key Laboratory for Green Chemical Technology of the Ministry of Education, School of Chemical Engineering and Technology, Tianjin University, Tianjin, China; ^4^Department of Material Science and Engineering, Institute of Space Science and Technology, Islamabad, Pakistan; ^5^Basic Science and Humanities Department, University of Engineering & Technology, Lahore, Pakistan; ^6^Department of Physics, University of Lahore, Gujranwala, Pakistan

**Keywords:** oxygen evaluation reaction, PT, nanosheeet, Mn_2_O_3_, nitrogen doped carbon

## Abstract

The purpose of this study is to develop an active, low cost, non-precious, stable, and high-performance catalyst for oxygen reduction reaction (ORR). In this regard, Mn_2_O_3_-decorated nitrogen-doped carbon nanosheets (Mn_2_O_3_/NC) are fabricated by a two-step strategy involving a hydrothermal method and a solid-state method. In the resultant structures, very fine Mn_2_O_3_ nanoparticles with an average size of about 5 nm are strongly attached to nitrogen-doped carbon nanosheets. The role of the Mn_2_O_3_ nanoparticles is to provide active sites for ORR, while the presence of the nitrogen-doped carbon not only enhances the conductivity of the overall structure but is also helpful for overall stability. The Mn_2_O_3_/NC shows good onset potential (0.80 V@−1 mA/cm^2^), methanol crossover effect, and stability (90%).

## Introduction

Over the past 100 years, global energy intensity has declined continuously due to economic and population growth. World energy consumption is projected to rise by 2.3% per year (Lewis and Nocera, 2006). It is estimated that energy demand will nearly double by 2050 (Du and Eisenberg, 2012). Eighty percent of the current energy resources are from fossil fuels (Ran et al., 2014), but the use of fossil fuels is associated with numerous problems like unsustainably, finite reserves (Dincer, 2000), buildup of greenhouse gases, and the emission of CO_2_ (Ran et al., 2014). To reduce the dependency on fossil fuels and to meet global energy demand in a sustainable fashion, renewable energy sources like fuel cells, metal-air batteries, and water splitting are important (Dincer, 2000; Du and Eisenberg, 2012; Ran et al., 2014). These energy systems are governed through electrochemical reactions in which oxygen reduction reaction (ORR) is particularly important (Dincer, 2000; Du and Eisenberg, 2012; Ran et al., 2014).

ORR occurs at the cathode of fuel cells, but its kinetics are six times slower than the anode reaction (Post, 1999; Linda et al., 2000; Pokropivny, 2007; Jingjing et al., 2014; Augustin et al., 2015; Jiao et al., 2015; Wei et al., 2015; Deng et al., 2016; Liu et al., 2016; Lv et al., 2016; Sun et al., 2016; Zhu et al., 2016). Therefore, a catalyst is required to enhance the speed of ORR. In this regard, Platinum (Pt) holds great importance due to its high efficiency and low onset potential (Linda et al., 2000). However, its high cost, rarity, and low stability are major concerns for the above-mentioned energy technologies (Hu and Dai, 2016). Thus, the development of a stable, active, and low-cost ORR catalyst is required in order to replace Pt (Lewis and Nocera, 2006).

Transition metals oxides (MnO, CoO, NiO) are considered to be the best alternative to noble metals for ORR. In particular, Mn_2_O_3_, because of the diversity of its oxidation states (2^+^, 3^+^, 4^+^), shows remarkable catalytic activity toward ORR (Jiao et al., 2015). Mn_2_O_3_ has the advantages of low cost, non-toxicity, compatibility with the environment, and abundant reserves (Lv et al., 2016). It also has various structural configurations (Wei et al., 2015). All these aspects of Mn_2_O_3_ make it an ideal candidate for ORR. However, there are still some issues that need to be resolved such as its low conductivity (Jiao et al., 2015).

On the other hand, because of availability, low price, and variety of form, carbon-based electro-catalysts have attracted much intention over the last few years. They show excellent ORR activities, comparable and even superior to that of Pt (Hu and Dai, 2016). Carbon-based nanostructures are also used to increase the conductivity for ORR. Carbon nanosheets and nanotubes are mostly used in this regard (Post, 1999; Jingjing et al., 2014). They also increase the surface area, and hence the number of active sites. However, pure carbon has low or moderate electrocatalytic activity (Jingjing et al., 2014; Liu et al., 2016). In order to improve the electrocatalytic activity of carbon nanostructures, different strategies are used, such as doping and combining them with other materials. Doping with heteroatoms like nitrogen (N) in carbon (C) is valuable because the electron affinity of N is greater than that of C; the carbon atoms adjacent to nitrogen dopants counterbalance this electron affinity by creating a net positive charge density. N doping attracts electrons to facilitate the ORR (Deng et al., 2016). It increases not only the electrocatalytic activity (Sun et al., 2016) but also the stability.

Keeping the aforementioned aspects in mind, the fabrication of Mn_2_O_3_ on NC may be a good approach to enhancing the activity and stability of ORR. The small Mn_2_O_3_ particle provides a larger surface area. NC sheets not only provide the support but also increase the conductivity and number of electrons transferred. Herein, we have fabricated Mn_2_O_3_-decorated nitrogen-doped carbon nanosheets (Mn_2_O_3_/NC) by using hydrothermal and solid-state methods. The resultant Mn_2_O_3_/NC has very small, ~5-10 nm Mn_2_O_3_ nanoparticles, providing a large surface area and number of active sites, while the nitrogen-doped carbon nanosheets increase not only the conductivity but also the stability of ORR. We explored the ORR activity of Mn_2_O_3_ supported by NC at different temperatures and concentrations and found that Mn_2_O_3_/NC at 500°C and with a 20% initial Mn_2_O_3_ concentration (Mn_2_O_3_/NC-500-20%) shows the best ORR performance, with low onset potential and excellent stability along with a good methanol crossover effect.

## Results and Discussion

**Figure 1:**
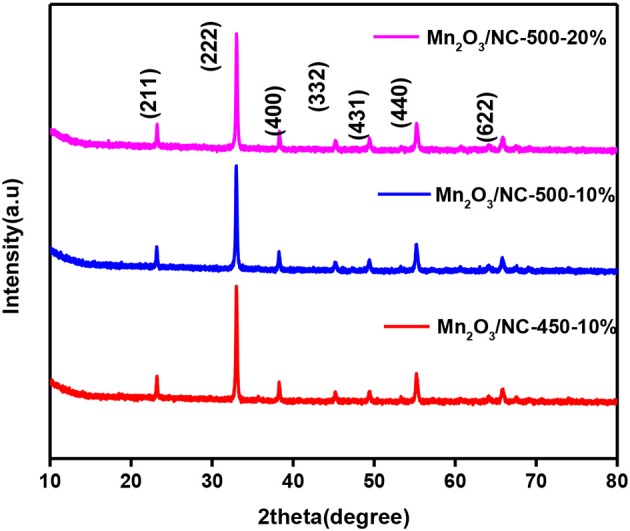
XRD patterns of Mn_2_O_3_/NC500-20%, Mn_2_O_3_/NC-500-10%, and Mn_2_O_3_/NC-450-10%.

In XRD, the Mn_2_O_3_ phase was identified with card number 41-1442. From XRD analysis, it can be seen that Mn_2_O_3_ is in the cubic phase with a=b=c=9.04 Å. No other peaks are observed, which confirms the high purity of the material, as stronger and sharper peaks indicate the formation of a well-defined crystalline structure. In order to see the effect of temperature and initial concentration on the XRD pattern, we compare the XRD patterns of three different samples, i.e., Mn_2_O_3_/NC-500-20%, Mn_2_O_3_/NC-500-10%, and Mn_2_O_3_/NC-450-10%. As shown in Figure 1, there is little difference in the intensities of the Mn_2_O_3_ nanoparticle XRD peaks for the fabricated three samples, which means that their Mn_2_O_3_ particle sizes are similar.

**Figure 2:**
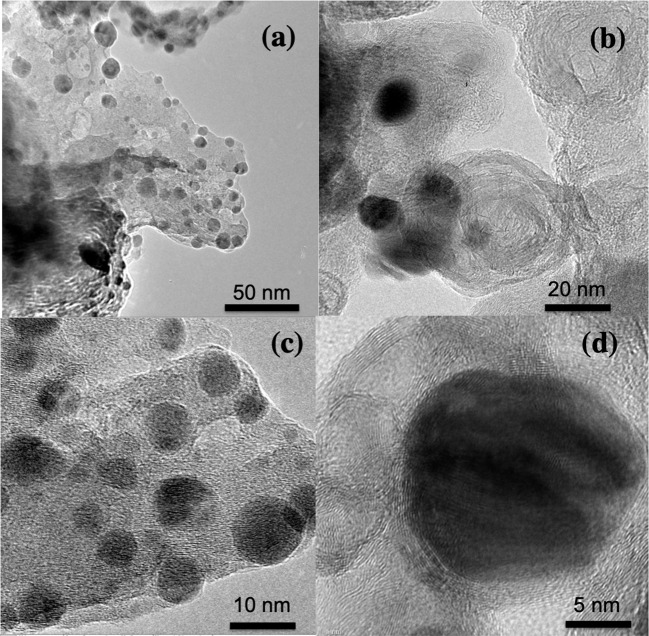
**(a-d)** TEM images of Mn_2_O_3_/NC-500-20 at different resolutions.

Mn_2_O_3_ nanoparticle-decorated nitrogen-doped carbon nanosheets (Mn_2_O_3_/NC) were fabricated through the pyrolysis of Mn(OH)_2_ and nitrogen-rich compounds, namely urea, in a muffle furnace. During the pyrolysis process, NH_3_ released from urea reduces Mn(OH)_2_ into Mn_2_O_3_ nanoparticles at the same time as Mn_2_O_3_ catalyzes the carbonization of carbon to form N-doped carbon nanosheets. SEM was used to determine the microstructures and morphology of the Mn_2_O_3_/NC. The SEM results for Mn_2_O_3_/NC-450-20%, Mn_2_O_3_/NC-500-20%, Mn_2_O_3_/NC-450-10%, and Mn_2_O_3_/NC-500-10% are shown in Figures S1–S4. SEM images of samples Mn_2_O_3_/NC-500-2.5%, Mn_2_O_3_/NC-500-5%, Mn_2_O_3_/NC-500-10%, and Mn_2_O_3_/NC-500-20%, which have initial concentrations of Mn(OH)_2_ of 2.5, 5, 10, and 20% and were fabricated at 500°C, are shown in Figure S5. We used TEM to image the nanostructures more clearly. The TEM images of a prepared sample in Figure 2 show that Mn_2_O_3_ particles are embedded in nitrogen-doped carbon nanosheets. Very thin nanosheets of carbon can be seen in all of the TEM images, while very small ~5–10 nm nanoparticles of Mn_2_O_3_ (black dots) can be observed on the surface of the NC.

Electrochemical measurement was carried out in a three-electrode electrochemical cell. Rotating disk electrode (RDE) measurements were performed to investigate the ORR activities in 0.1 M KOH solutions. Hg/HgO was used as a reference electrode, and platinum wire was used as a counter electrode. A glassy carbon disc with a diameter of 2.5 mm served as a substrate for the working electrode.

**Figure 3:**
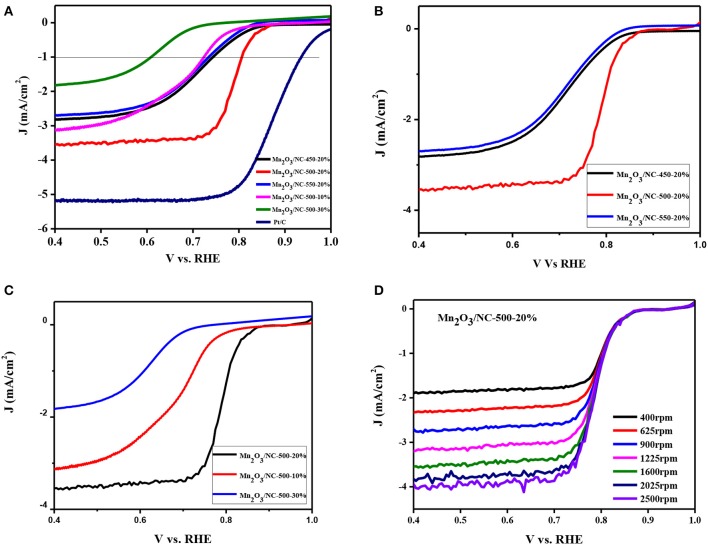
LSV curves obtained in oxygen-saturated 0.1 M KOH electrolyte at a 0.80 V sweep rate. **(A)** Comparison of ORR activities of different samples demonstrates that a temperature of 500°C and an initial Mn(OH)_2_ concentration of 20% is the best combination; **(B)** the effect of temperature on ORR activity; **(C)** the effect of concentration of Mn(OH)_2_ on ORR activity; **(D)** LSVs of Mn_2_O_3_/NC-500-20 in O_2_-saturated 0.1 M KOH solution at different rotation speeds of 400–2,500 rpm.

The results of linear sweep voltammetry (LSV) of samples Mn_2_O_3_/NC-450-20%, Mn_2_O_3_/NC-500-20%, Mn_2_O_3_/NC-550-20%, Mn_2_O_3_/NC-500-10%, and Mn_2_O_3_/NC-500-30% at 1,600 rpm in 0.1 M KOH and Pt/C are shown in Figure 3A. The onset potentials of all the tested samples at a current density of −1 mA/cm^2^ are given in Table 1.

**Table 1:** Onset potential @−1 mA/cm^2^ for all samples.

**Sample name**	**Onset potential**
Mn_2_O_3_/NC-500-30%	0.6079
Mn_2_O_3_/NC-500-10%	0.72385
Mn_2_O_3_/NC-550-20%	0.73211
Mn_2_O_3_/NC-450-20%	0.74144
Mn_2_O_3_/NC-500-20%	0.80462
Pt/C	0.933

Figure 3A and Table 1 show that Mn_2_O_3_/NC-500-20% has the best onset potential (0.732 V @-1mA/cm^2^) of the fabricated samples of Mn_2_O_3_/NC. However, the onset potential of Pt/C (0.93 V @−1mA/cm^2^) is much better than that of Mn_2_O_3_/NC-500-20%. Furthermore, we conclude from our results that fabrication at 500°C and an initial concentration of 20% Mn(OH)_2_ are the best approximation to the performance of Pt/C. Three samples (Mn_2_O_3_/NC-450-20%, Mn_2_O_3_/NC-550-20%, and Mn_2_O_3_/NC-500-20%) with the same initial concentration of 20% but with different fabrication temperatures are compared in Figure 3B. At high temperature, the organic species are evaporated from the sample (all organic groups such as C will be removed on heating). This loss of organic groups occurs due to the evolution of CO and CO_2_ gases and the decomposition of organic ligands by oxidation (Pokropivny, 2007). This is dependent on temperature and the partial pressure of oxygen (Pokropivny, 2007). Therefore, with the decrease in C, Mn_2_O_3_ particles become active or the amount of Mn_2_O_3_ will increase. When Mn_2_O_3_ becomes active, it can lose oxygen. The loss of oxygen will result in the generation of vacancies on the surface. Both the electrical and chemical properties and the performance of the catalyst and its support change because of the presence of such oxygen vacancies (Linda et al., 2000). When Mn_2_O_3_ nanoparticles are more accessible to oxygen due to these vacancies, a large triple-phase boundary will result, which further facilitates ORR.

In order to achieve better ORR activity, there should be a balance in the coverage of the surface by oxygenated species, which are specifically anions. These anions are adsorbed on the catalyst surface and have intermediate adsorption energies. Therefore, if the catalyst binds oxygen atoms too strongly, the ORR process will slow down due to the slow rate of removing oxides and anions from the surface. Conversely, if the catalyst binds oxygen atoms too weakly, the ORR process will be limited by the rate of electron and proton transfer to adsorbed O_2_ (Augustin et al., 2015). Thus, the catalyst may bind oxygen too strongly at 450°C and too weakly at 550°C, so that both conditions produce Mn_2_O_3_/NC that is less active than with a temperature of 500°C. In addition, through our experiment, we can see that at 550°C, a large amount of the sample evaporates or vanishes, so with the increase in temperature above 550°C, the ORR activity will decrease.

The activities of samples Mn_2_O_3_/NC-500-10%, Mn_2_O_3_/NC-500-20%, and Mn_2_O_3_/NC-500-30%, all with the same temperature condition of 500°C, are compared in Figure 3C. It can be concluded that the low number of Mn_2_O_3_ particles where the concentration is 10% means that they cannot take part in the reaction properly and that a concentration of 30% means that the amount of Mn_2_O_3_ particles is too high, and there is therefore insufficient carbon support. Thus, a concentration of 20% is the best option on the basis of our results, showing a balance between the amounts of carbon and Mn_2_O_3_.

The working electrode was scanned with RDE voltammograms for ORR at different rotation speeds. Linear-sweep voltammograms (LSVs) were obtained of Mn_2_O_3_/NC-500-20% in an O_2_-saturated 0.1 M KOH solution at different rotation speeds of 400–2500 rpm. The current density for the LSV curves increased with increasing rotation speed due to the increase in mass transport on the electrode surface, as shown in Figure 3D.

**Figure 4:**
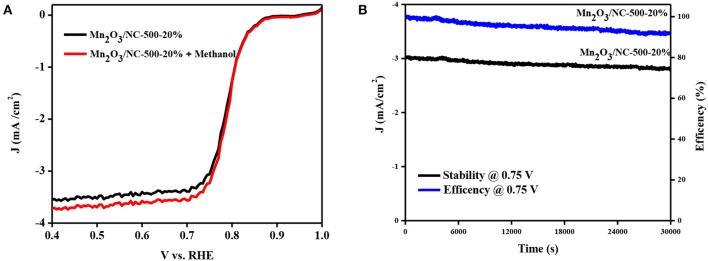
**(A)** LSV of sample Mn_2_O_3_/NC-500-20 with and without methanol. **(B)** Current–time (i–t) chronoamperometric response of Mn_2_O_3_/NC-500-20 for 30,000 at 0.75 V.

For practical applications in fuel cells, the methanol crossover effect and stability are both important parameters. The methanol crossover effect was investigated by LSV measurements in 0.1 M KOH with the addition of methanol to Mn_2_O_3_/NC-500-20%. There is only a minor change in the LSV curves for ORR under the addition of methanol to Mn_2_O_3_/NC-500-20%. This shows a good methanol tolerance, with improved ability to avoid cross effects (Figure 4A). A stability test was conducted for Mn_2_O_3_/NC-500-20%. using the i-t chronoamperometric response at a constant voltage of 0.75 V in a 0.1 M KOH solution at 1,600 rpm. Mn_2_O_3_/NC-500-20% shows a kinetic current density of about 91% after 30,000 s. The efficiency of sample Mn_2_O_3_/NC-500-20% after 30,000 s remains 73%. These results, shown in Figure 4B, demonstrate that Mn_2_O_3_/NC-500-20% has better long-term durability.

In conclusion, we fabricated nitrogen-doped carbon nanosheets decorated with Mn_2_O_3_ nanoparticles by adopting a novel two-step synthesis method. The phase was identified as crystalline Mn_2_O_3_ with card number 41-1442. The particle size, calculated from TEM images, was ~5–10 nm. Mn_2_O_3_/NC-500-20% exhibited good ORR performance with low onset potential (0.732 V@-1 mA/cm^2^), excellent stability, and a good methanol crossover effect. This low cost and easy fabrication method of Mn_2_O_3_/NC can be used for large-scale fabrication not only of Mn_2_O_3_ but also of other transition metal oxide/NCs. Because of its good stability and low onset potential, Mn_2_O_3_-500-20% can be used in fuel cells and metal-air batteries.

## Experimental Methods

### Fabrication of Mn_2_O_3_

Two-step synthesis, involving a hydrothermal method and a solid-state method, was used.

**Scheme 1:**
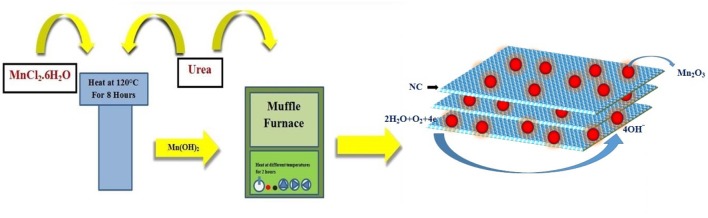
Flow chart of fabrication.

Briefly, a solution of MnCl_2._6H_2_O and urea is stirred for 20 min in 20 mL of distilled water. The resulting solution is transferred to a Teflon container and placed in an autoclave. This autoclave process is carried out for 8 h at 120°C. The solution is then washed several times with distilled water and ethanol in a centrifuge machine; finally, it is dried at 60°C for 8 h. After drying, this intermediate product is mixed with urea and exposed to different temperatures in a muffle furnace for 2 h. The details of the process are given in Scheme 1.

### Characterization of the Materials

According to the elaboration procedure, the Mn_2_O_3_ nanoparticles are supported by NC. A scanning electron microscope (SEM) was used for the morphological analysis. We investigated the particle size distribution using transmission electron microscopy (TEM). X-ray Diffraction (XRD) was used in order to investigate the crystal structures of Mn_2_O_3_/NC.

### Electrochemical Setup

The electrochemical measurements were carried out in a three-electrode cell. Rotating disk electrode (RDE) measurements were performed to investigate the ORR activities in 0.1 M KOH. Hg/HgO was used as a reference electrode, and platinum wire was used as a counter electrode. A glassy carbon disc with a diameter of 2.5 mm served as a substrate for the working electrode.

## Data Availability Statement

All datasets generated for this study are included in the article/Supplementary Material.

## Author Contributions

MQas has written the manuscript. MQad has done the experimental work. JH, SB, and MHF helped in characterization. JZ has provided the experimental facilities. MTah has designed and supervised the overall work. FI and MTan have revised the manuscript while MQF helped in experimentation.

### Conflict of Interest

The authors declare that the research was conducted in the absence of any commercial or financial relationships that could be construed as a potential conflict of interest.
