# Supplementary material for: Nitrogen-Doped Carbon Nanosheets Decorated With Mn2O3 Nanoparticles for Excellent Oxygen Reduction Reaction
Source: Front Chem. 2019 Nov 7;7:741. doi: 10.3389/fchem.2019.00741 (PMC6856643; doi:10.3389/fchem.2019.00741)
Supplement: Supplementary file 1 [file Table_1.DOCX]

**Supporting Information**

**For**

**Nitrogen doped carbon nanosheets decorated with Mn_2_O_3_ nanoparticles for excellent Oxygen Reduction Reaction**

**Experimental Section**

*Fabrication of Mn_2_O_3_/NC*

Two step synthesis involving hydrothermal method and solid-state method is used.

Briefly, solution of MnCl_2._6H_2_O and urea is stirred for 20 minutes in 20mL of distilled water. The resulting solution is transferred to Teflon container and placed in autoclave. This autoclave process is carried out for 8 hours at 120^o^C. Then the solution washed several times with distilled water and ethanol in centrifuge machine and dried at 60^o^C for 8 hours. After drying, this intermediate product is mixed with urea and exposed at different temperatures in muffle furnace for 2 hours.

*Characterization of the materials*

According to the elaboration procedure (see section 3.1) the Mn_2_O_3_ nanoparticles are supported onto NC. For the morphological analysis, scanning electron microscope (SEM) is used. We investigated their particle size distribution using transmission electron microscopy (TEM). In addition, X-ray Diffraction (XRD) experiments have been
undertaken for the three Mn_2_O_3_/NC-based materials.

*Electrochemical Setup*

The electrochemical measurements were carried out in conventional three-electrode cell. Rotating disk electrode (RDE) measurements were performed to investigate the ORR activities. We tested activities for ORR in alkaline media. In alkaline media, 0.1 M KOH solutions are used. Hg/HgO was used as a reference electrode and platinum plate was used as a counter electrode. A glassy carbon disc with diameter 2.5mm served as a substrate for working electrode.

## Morphological analyses

**Figure S1** shows the SEM images of **Mn_2_O_3_/NC-450-20%** at 450°C with 20% initial concentration of Mn (OH) _2_. It can be seen that particles are in the form of groups and combined together to form cubical morphology with some pores. Agglomerations with numerous small particles are formed.


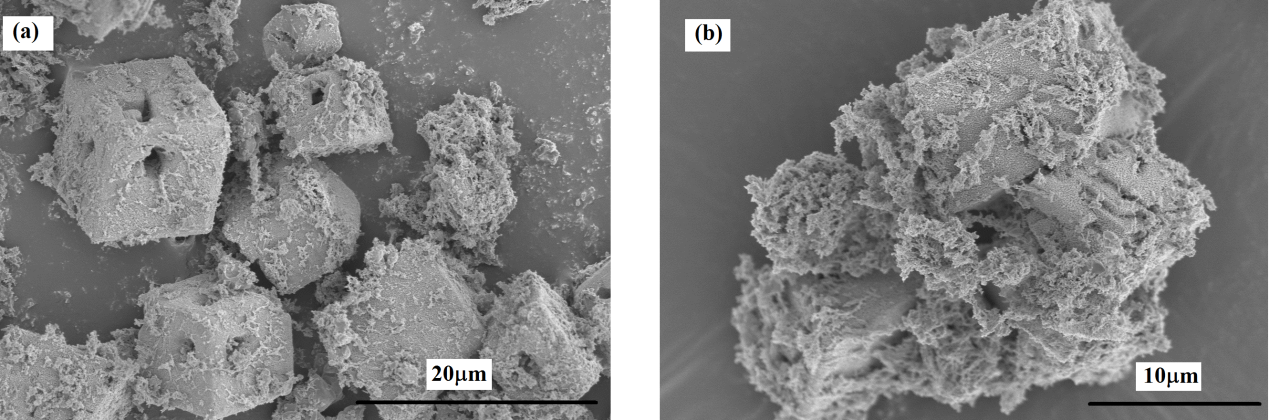


**Figure S1**: (a, b) SEM images of **Mn_2_O_3_/NC-450-20** with 20% initial concentration of Mn(OH)_2_ at 450°C.

**Figure S2** shows the SEM image of sample **Mn_2_O_3_/NC-500-20** at 500°C with 20% initial concentration of Mn (OH) _2_. SEM images show agglomeration of nanoparticles, these particles aggregate together and form larger group of cube shaped particles.


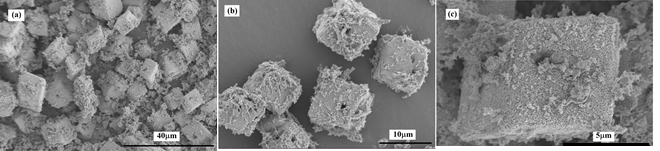


**Figure S2**: (a-c) SEM image at 500°C with 20% initial concentration of Mn (OH) _2_.

**Figure S3** shows the SEM image of **Mn_2_O_3_/NC-450-10** at 450°C with 10% initial concentration of Mn (OH) _2_.


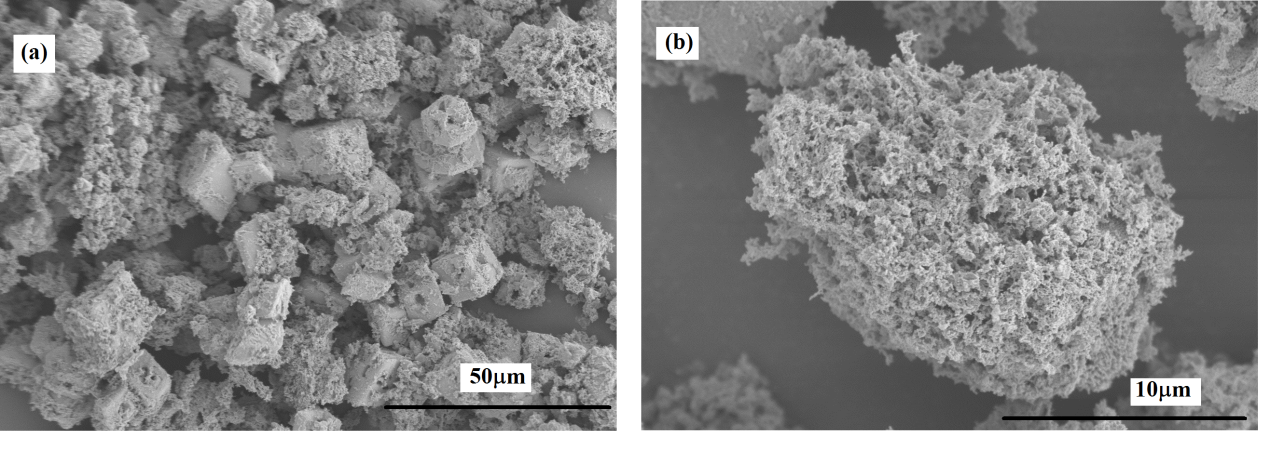


**Figure S3:** (a, b) SEM images at 450°C with 10% initial concentration of Mn (OH) _2_.

**Figure S4** shows the SEM images of **Mn_2_O_3_/NC-500-10** at 500°C with initial concentration of 10%. They formed a network like structure. Nanostructures formed by particles are not uniform.


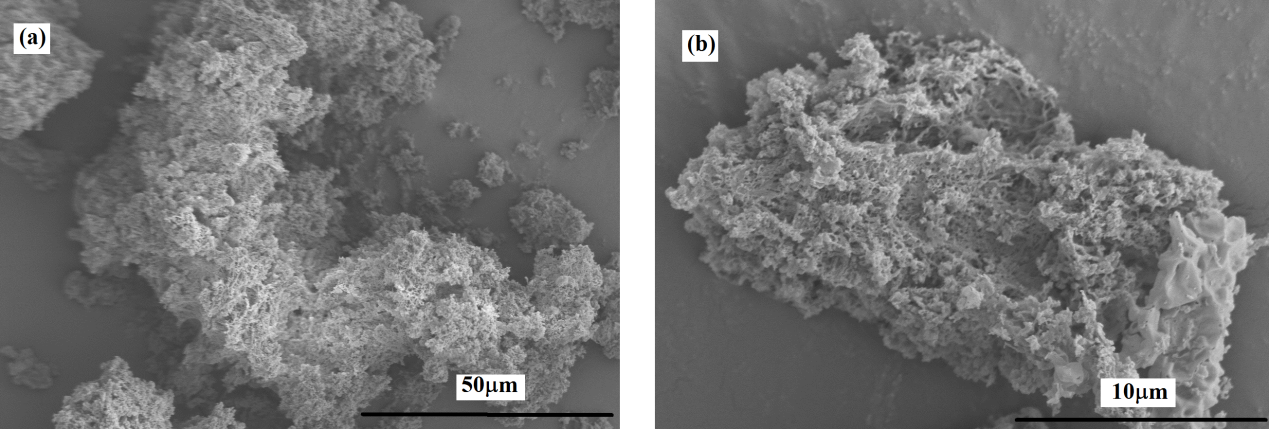


**Figure S4:** (a, b) SEM image at 500°C with initial concentration of 10%.

**Figure S5** shows the SEM images at **500°C** temperature with different initial concentration of Mn (OH) _2_. Figure S5(a), (b), (c) and (d) shows SEM images of samples **Mn_2_O_3_/NC-500-2.5, Mn_2_O_3_/NC-500-5, Mn_2_O_3_/NC-500-10** and **Mn_2_O_3_/NC-500-20** with 2.5%, 5%, 10% and 20% initial concentration of Mn (OH) _2_ at 500°C respectively.


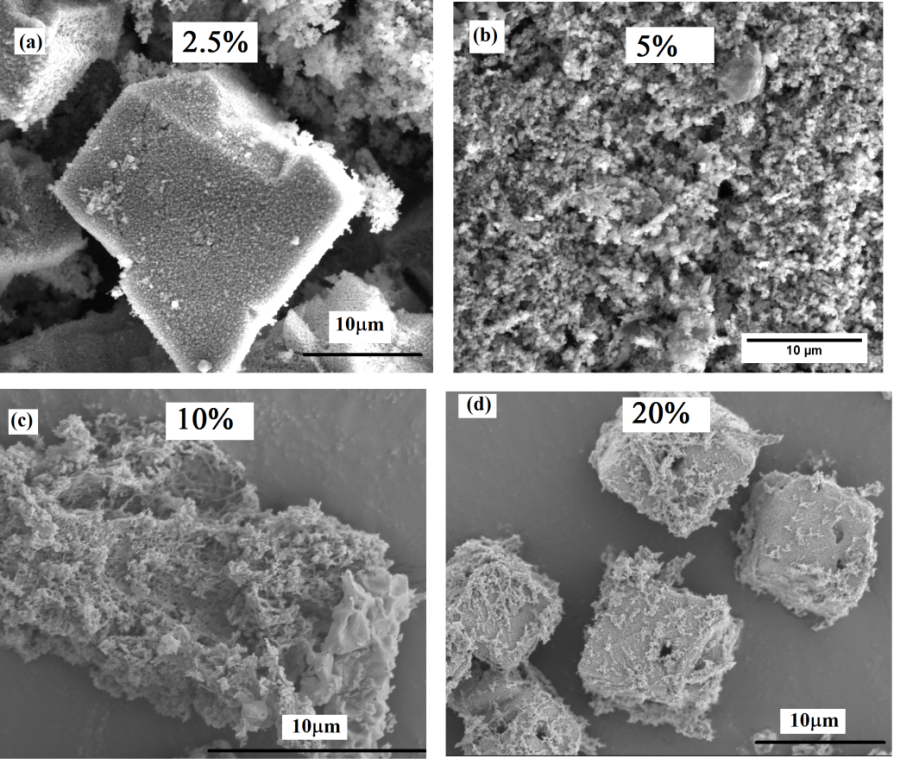


**Figure S5**: (a), (b), (c) and (d) SEM images at 500°C with 2.5%, 5%, 10% and 20% initial concentration of Mn(OH)_2_ respectively
